# Urinary iodine excretion and thyroid function status in school age children of hilly and plain regions of Eastern Nepal

**DOI:** 10.1186/s13104-015-1359-6

**Published:** 2015-08-26

**Authors:** Prem Raj Shakya, Basanta Gelal, Binod Kumar Lal Das, Madhab Lamsal, Paras Kumar Pokharel, Ashwini Kumar Nepal, David A. Brodie, Gauri Shankar Sah, Nirmal Baral

**Affiliations:** Department of Biochemistry, School of Medicine, Patan Academy of Health Sciences, Lagankhel-5, PO Box: 26500, Lalitpur, Nepal; Department of Biochemistry, B.P. Koirala Institute of Health Sciences, Dharan, Nepal; Department of Community Medicine, B.P. Koirala Institute of Health Sciences, Dharan, Nepal; Department of Biochemistry, Faculty of Society and Health, Bucks New University, High Wycombe, Buckinghamshire UK; Department of Paediatrics and Adolescent Medicine, B.P. Koirala Institute of Health Sciences, Dharan, Nepal

**Keywords:** Iodine status, Iodized salt, Thyroglobulin, Thyroid function, Nepal

## Abstract

**Background:**

Iodine deficiency is a major public health problem in many developing countries including Nepal. The present study was designed to investigate the urinary iodine excretion (UIE), thyroid function status and household salt iodine content (SIC) in school-aged children (SAC) and to establish the relationships between these factors.

**Methods:**

A community-based cross sectional study was conducted in selected schools of two districts, Tehrathum and Morang, lying in the hill and plain region of eastern Nepal respectively. A total of 640 SAC, (Tehrathum n = 274 and Morang n = 366) aged 6–11 years, were assessed for UIE and household SIC. Among the 640 children, 155 consented to blood samples (Tehrathum n = 78 and Morang n = 77) to test for serum thyroglobulin (Tg), thyroid stimulating hormone (TSH), free triiodothyronine (fT_3_) and free thyroxine (fT_4_). UIE was measured by ammonium persulfate digestion method. SIC was measured by iodometric titration method and Tg, TSH, fT_4_ and fT_3_ were measured by immunoassay based kit method.

**Results:**

In Tehrathum and Morang, 9.5 and 7.7 % of SAC had UIE values of UIE <100 µg/L while 59.5 and 41 % had iodine nutrition values of >299 µg/L, with median UIE of 345.65 and 270.36 µg/L respectively. The overall medians were as follows, Tg 14.29 µg/L, fT_3_ 3.94 pmol/L, fT_4_ 16.25 pmol/L and TSH 3.61 mIU/L. There was a negative correlation between UIE and Tg (r = −0.236, p = 0.003) and a positive correlation between UIE and SIC (r = 0.349, p < 0.0001). We found 19.5 %, n = 15 and 16.7 %, n = 13 subclinical hypothyroid cases in Morang and Tehrathum respectively. Iodometric titration showed only 6.4% (n = 41) of the samples had household SIC <15 ppm. Multivariate analysis revealed that use of packaged salt by SAC of Tehrathum district correlated with higher UIE values.

**Conclusions:**

Our focused data suggests that collaborative universal salt iodization (USI) programs are improving the health of children in the Tehrathum and Morang districts of Nepal. We also found that excessive iodine in a large portion of the study groups is a substantial concern and iodine intervention programs need to deal with both deficient and excessive iodine scenarios that can both be present simultaneously in study populations.

## Background

Iodine deficiency (ID) is a major public health problem globally, including Nepal [1]. It affects the population of all age groups but neonates, infants, pregnant women and school children constitute the most vulnerable groups [2]. Although remarkable progress has been made in the control of iodine deficiency disorders (IDD), they still remain a significant global public health problem particularly in developing countries [3]. The most severe outcomes of ID are increased perinatal mortality and mental retardation. ID is considered as the most common cause of brain damage in childhood which can be prevented, if appropriate measures are taken sufficiently early [4, 5]. Thyroid hormone plays a critical role during brain development [6]. The most common thyroid disorders in areas of excess iodine intake are autoimmune thyroid diseases, nodular goiter and iodine-induced hyperthyroidism [7, 8].

The World Health Organization (WHO) estimated the worldwide prevalence of ID using urinary iodine excretion (UIE) data sampling 92 % of the world’s population from 1993 to 2003 [4]. Nearly two billion individuals have inadequate iodine status (UIE <100 µg/L). Occurrence of ID was observed in 36.4 % of school-aged children (SAC). Various indicators are used to investigate the iodine status of a population: thyroid size, urinary iodine content, blood levels of thyroid stimulating hormone (TSH), and serum thyroglobulin (Tg) [9, 10] although all of these indicators have some limitations.

Urinary iodine is a sensitive indicator of recent iodine intake [11, 12], but not of thyroid function. Since most of the iodine that is absorbed is excreted in the urine, the urinary iodine level is a good marker of a recent dietary intake. Spot urine sample is recommended for population based studies [13, 14]. Thyroid size measurement reflects a population’s history of iodine nutrition, but not present iodine status [15]. It may not return to normal size for months or years after correction of ID; thus, it is not a good indicator of IDD after introduction of iodized salt [9]. TSH level in neonates are particularly sensitive to ID, although the cost of implementing a TSH screening program is too high for most developing countries [16]. Tg, the most abundant thyroid specific protein, with no known physiologic role outside the thyroid [17], is a primary precursor in the production of thyroid hormones. Tg assay has recently been adapted for use on dried blood spots (DBS) and the normal reference range for SAC has been established [17]. The value of Tg as an indicator of global IDD status has yet to be fully explored [4], but as the results from population studies indicate, Tg seems to be a valuable indicator of thyroid status in respect to its sensitivity to recent changes in iodine status [9]. An adequate iodine intake may be assumed when TSH and Tg values are at the lower end of the normal range [18]. In iodine-deficient populations, serum fT_3_ may be increased or remains unchanged, and serum fT_4_ usually declines [19, 20]. But, these changes often lie within the normal range, and appear similar with iodine-sufficient individuals, which makes thyroid hormone levels a nonspecific measure of iodine status [16].

Iodized salt has been considered as the most effective tool to control and prevent IDD. Universal salt iodization (USI) has been significantly successful in most countries [21]. The goal of USI (>90 % households using adequately iodized salt) has already been achieved by many countries while others have not yet reached these levels [9, 16]. Delivery of iodized salt is sporadic, especially in remote areas, and the salt is often left outside, thus causing a rapid loss of iodine in inclement weather [9]. Recent data indicate that ID is still a significant public health problem in Nepal, with 19.4 % SAC having UIE <100 µg/L [1, 22]. Thus, this study aims to determine the current iodine and thyroid status in school children in the Tehrathum and Morang districts of Eastern Nepal by measuring UIE, serum Tg, fT_3_, fT_4_, TSH and salt iodine content (SIC). The study will also determine the relationships between these measures to help guide future studies to measure the incidence of IDD efficiently.

## Methods

### Study sites and participants

This cross sectional study was designed to study iodine nutrition and thyroid status of randomly selected schools in Tehrathum district, situated in the hilly region and Morang district lying in the plains region of Eastern Nepal. It used the iodine status indicators namely UIE, serum Tg and SIC, which are recommended by WHO, UNICEF and the International Council for Control of Iodine Deficiency Disorders (ICCIDD) [16]. We have classified the iodine status of median UIE according to epidemiological criteria developed by WHO, UNICEF and ICCIDD for SAC (≥6 years) as severe ID (<20 µg/L), moderate ID (20–49 µg/L), mild ID (50–99 µg/L), adequate iodine nutrition (100–199 µg/L), above requirements (100–199 µg/L) and excessive (>300 µg/L) [16]. The study was conducted over 13 months (from May 2010 to June 2011). A total of seven primary schools, three from Tehrathum and four from Morang were selected using random number table from the list of all the schools in these districts separately, as a primary sampling unit. Then all the children aged 6–11 years in these schools were included as the ultimate sampling unit. This sampling design was equivalent to conducting a 30 × 20 cluster sampling (600 children) as the average number of children aged 6–11 years in each school was found to be around 90 (630 children). A total of 640 urine and salt samples from the children’s households were collected and screened for UIE and SIC. Out of 640 children, blood samples from 155 children with parental consent were collected for the estimation of serum TSH, fT_3_, fT_4_ and Tg.

### Ethical clearance

Prior visits were made to the respective schools; the objectives of the study and its benefits were explained to the school authorities and children’s guardians. The ethical clearance for this study was approved by the Institutional Ethical Review Board (IERB) of B.P. Koirala Institute of Health Sciences (BPKIHS), Dharan, Nepal and included permission to obtain urine and blood samples from SAC 6–11 years. Written consent was obtained from guardians of the children and verbal assent was taken from children.

### Sample collection and analysis

Spot urine samples (n = 640) were collected from all the eligible children in a clean sterile pre-labeled tightly screw capped 15 mL plastic vials. Clean, airtight plastic pouches were distributed to the same eligible children with clear instructions for collecting the salt samples from their homes, which were collected the following day. Every effort was made to ensure that urine samples were not contaminated. The children went to the toilet individually so that urine could not be ‘shared’. Blood samples (3 mL) were collected by venipuncture in a plain red capped vacutainer (BD vacutainer, USA) and transported to the biochemistry laboratory at BPKIHS, maintaining a cold chain. The blood was centrifuged immediately at 3000 g for 10 min, and serum was separated into two aliquots and stored at −20 °C until analysis. Batch analyses were performed with calibrators and standards. Urinary iodine levels were assessed by the Ammonium Persulfate Digestion Microplate (APDM) method using the Sandell-Kolthoff reaction in a specially designed apparatus, sealing cassette (Hitachi, Japan) [23] in microplate format. Urinary iodine controls L_1_ (72–96 µg/L) and L_2_ (260–348 µg/L) (Seronorm, Norway) were analyzed to obtain intra-assay CVs (L_1_ = 7.4 %, L_2_ = 3.3 %) and inter assay CVs (L_1_ = 23.5 %, L_2_ = 11.26 %) respectively. Measurement of Tg was undertaken using a standard kit (Genesis Diagnostics Ltd, UK) [24] whereas TSH, fT_3_ and fT_4_ analyses were done using a HUMAELISA kit (Human^®^ Diagnostics, Germany). Thyroid function status was determined according to the biochemical criteria taking consideration of the reference range provided in the kit, which reported fT_3_ 2.16–6.47 pmol/L, fT_4_ 10.32–28.38 pmol/L, TSH 0.39–6.16 mIU/L and Tg 3–50 µg/L. Subclinical hypothyroidism are considered when TSH >6.16 mIU/L with fT_3_ and fT_4_ in the reference range whereas in subclinical hyperthyroidism, TSH <0.39 mIU/L with fT_3_ and fT_4_ in the reference range.

### Statistical analysis

Data were first entered in Microsoft Excel™ 2010 and then converted to Statistical Package for Social Sciences (SPSS) version 16.0 (SPSS Inc., Chicago, USA). Data were first checked for the normality using Kolmogorov–Smirnov (KS) test. For normally distributed data, Pearson’s correlation test and the unpaired student ‘t’ test were applied. If data were not normally distributed, then either they were log transformed or non-parametric tests were applied. Multiple linear regression analysis was performed to find the main predictor of UIE among SAC. A p value of less than 0.05 was considered statistically significant at 95 % confidence interval (CI).

## Results

### Biochemical characteristics

#### Urinary iodine excretion (UIE)

**Fig. 1:**
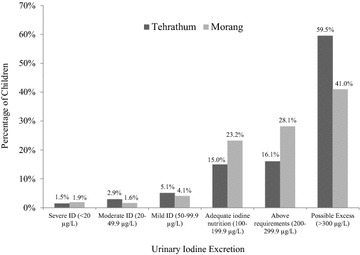
Percent distribution of urinary iodine concentrations in primary school children in the Tehrathum and Morang districts

The median UIE values of single spot urine tests are presented in Table 1 and results are reported as a population not individuals as suggested by Zimmerman et al. [14]. The overall median UIE is 291.8 µg/L (IQR 181.3–411.5 µg/L). Table 1 shows that boys were found to have a higher median UIE than girls but it was not statistically significant (p = 0.162). We found significant difference between age groups for UIE (p = 0.012). There were significant differences between 6–7 and 8–9 age categories for UIE (p = 0.004). Based on UIE, 1.7 % of SAC had severe, 2.2 % had moderate and 4.5 % had mild ID. High median values for UIE can be a cause for concern because this can have an adverse effect on health and is discussed more fully later. Figure 1 depicted the iodine status of the SAC of both Tehrathum and Morang districts based on the UIE.

**Table 1:** Urinary iodine concentration in relation to sex, age and location

	UIE (µg/L)			UIE category (µg/L)											
				Sever ID (<20)		Moderate ID (20–49)		Mild ID (50–99)		Adequate iodine nutrition (100–199)		Above requirements (200–299)		Excessive (>300)	
	n	Median	IQR	%	n	%	n	%	n	%	n	%	n	%	n
Total	640	292	181; 411	1.7	11	2.2	14	4.5	29	19.7	126	23	147	48.9	313
Gender^a^															
Male	312	301	196; 412	0.3	1	2.2	7	2.9	9	20.2	63	24.4	76	50	156
Female	328	285	163; 410	3	10	2.1	7	6.1	20	19.2	63	21.6	71	47.9	157
Age (years)^b^															
6–7	168	269	140; 390	4.2	7	3.6	6	6	10	25	42	19	32	42.3	71
8–9	226	320	209; 429	1.3	3	2.2	5	4.4	10	15	34	24.3	55	52.7	119
10–11	246	297	195; 411	0.4	1	1.2	3	3.7	9	20.3	50	24.4	60	50	123
District^a^															
Tehrathum	274	346	205; 463	1.5	4	2.9	8	5.1	14	15	41	16.1	44	59.5	163
Morang	366	270	167; 358	1.9	7	1.6	6	4.1	15	23.2	85	28.1	103	41	150

*UIE* urinary iodine excretion, *IQR* interquartile range, *ID* iodine deficiency

^a^Applied Mann–Whitney test

^b^Applied Kruskall–Wallis test, For Post Hoc (pairwise) comparison of age group, Mann–Whitney tests were applied

### Salt iodine content (SIC)

In Tehrathum, 34.7 % (n = 95) of the children consumed non-packaged salt in their household as compared to only 3.3 % (n = 12) in Morang, which is statistically significant (p < 0.0001). Morang had 98.6 % (n = 361) children with SIC >15 ppm as compared to Tehrathum 86.9 % (n = 238) in concordance with studies carried out in demographically and geographically similar regions [25, 26]. The mean SIC for Tehrathum and Morang by salt iodometric method was 39.4 (SD 18.0) and 43.3 (SD 12.0) ppm respectively. We found that SAC having UIE >100 µg/L was associated with packet salt consumption (p = 0.005). A larger percentage (94.5 %) of school children who consumed packet salt have been found to have UIE values >100 µg/L.

### Serum specimens

Mean, standard deviation and median with inter quartile range (IQR) of serum fT_3_, fT_4_, TSH and Tg in Tehrathum and Morang districts are summarized in Tables 2, 3. In this study, hypothyroidism or hyperthyroidism is classified by considering all three thyroid related hormones.

**Table 2:** Mean (SD), median with IQR of serum Tg, fT3, fT4 and TSH

	Tehrathum (n = 78)				Morang (n = 77)				Total (n = 155)			
	Mean	SD	Median	IQR	Mean	SD	Median	IQR	Mean	SD	Median	IQR
Tg (µg/L)	25.7	28.3	19.5	11.0; 29.0	14.5	12.5	10.9	6.6; 17.7	20.1	22.6	14.3	8.8; 25.3
fT3 (pmol/L)	4.0	1.1	3.7	3.4; 4.6	4.2	1.1	4.0	3.4; 4.9	4.2	1.1	4.0	3.4; 4.6
fT4 (pmol/L)	15.5	3.9	15.5	12.9; 18.1	18.1	3.9	16.8	15.5; 19.4	16.8	3.9	16.8	12.9; 19.4
TSH (mIU/L)	4.5	4.7	3.5	2.3; 4.9	4.4	2.8	4.1	2.5; 5.8	4.5	3.8	3.6	2.4; 5.5

Reference range: Tg (3–50 µg/L), TSH (0.39–6.16 mIU/L), fT3 (2.16–6.47 pmol/L), fT4 (10.32–28.38 pmol/L)

*Tg* serum thyroglobulin, *fT3* free triiodothyronin, *fT4* free thyroxine, *TSH* thyroid stimulating hormone

**Table 3:** Characteristics of Tg, TSH, fT4 and fT3 of different iodine status in primary school age children (n = 155) of Tehrathum and Morang districts

UIE category	Tg^a^ (µg/L)	fT3 (pmol/L)	fT4 (pmol/L)	TSH^a^ (mIU/L)
Severe ID (n = 11)				
Mean	32.6	4.3	15.3	5.8
SD	38.2	1.1	3.8	2.6
Median	18.1	3.8	14.1	6.7
IQR	12.8; 29.0	3.5; 5.7	12.9; 18.1	3.6; 7.5
Moderate ID (n = 14)				
Mean	39.5	4.4	16.2	4.1
SD	59.3	1.1	3.5	2.9
Median	21.0	4.0	17.0	3.3
IQR	12.5; 35.9	3.7; 4.5	14.2; 19.4	2.1; 5.9
Mild ID (n = 29)				
Mean	25.5	4.3	16.8	4.7
SD	16.0	1.1	4.3	2.2
Median	22.7	4.3	16.3	4.1
IQR	13.2; 31.1	3.2; 4.5	12.9; 19.4	3.5; 5.8
Adequate iodine nutrition (n = 126)				
Mean	15.3	4.1	17.2	3.8
SD	9.8	1.2	4.6	2.0
Median	13.7	4.0	16.6	3.5
IQR	9.1; 18.7	3.4; 4.9	12.9; 20.6	2.2; 5.1
Above requirements (n = 147)				
Mean	18.6	3.8	16.4	5.3
SD	14.4	1.1	4.2	6.4
Median	15.2	3.6	15.6	3.7
IQR	8.1; 25.1	3.1; 19.4	19.4; 12.9	2.3; 5.0
Excessive (n = 313)				
Mean	17.2	4.1	16.3	4.3
SD	18.1	1.1	3.9	3.9
Median	10.9	4.1	15.9	3.2
IQR	6.5; 22.8	3.4; 4.9	12.9; 19.4	2.4; 5.5

One way ANOVA test shows significant difference (p = 0.016) between Tg and UIE category. Post hoc analysis was carried out for Tg, fT3, fT4 and TSH by UIE categories separately. No statistically significant difference observed for other parameters (fT3, fT4 and TSH) with UIE category

Level of significance: * p < 0.05

*ID* iodine deficiency, *Tg* serum thyroglobulin, *fT3* free triiodothyronine, *fT4* free thyroxine, *TSH* thyroid stimulating hormone

^a^Non-normally distributed variables were log transformed

### Serum thyroglobulin (Tg)

**Fig. 2:**
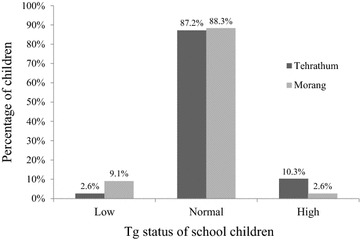
Serum Tg status in study subjects in Tehrathum and Morang districts. *According to the normal range (4–40 µg/L) provided by Zimmermann et al. [27]

The recently developed Tg assay for use on dried whole blood spots with CRM-457 Tg standards had a reference range (the 3rd and 97th percentiles, 5–14 year old children, euthyroid, anti TgAb negative, residing in long term iodine sufficiency) of 4–40 µg/L [27]. Accordingly, 9.5 and 1.9 % of the school children in the Tehrathum and Morang districts have Tg-based iodine deficiency [27]. The majority of the children’s Tg values fall in the normal reference range (See Fig. 2). Our result showed that median Tg concentration for Tehrathum and Morang was 19.5 µg/L (11.0–29.0 µg/L) and 10.9 µg/L (6.6–17.7 µg/L) respectively. We observed significant negative correlation between Tg and UIE in concordance with the study done by Zimmermann et al. [17, 28].

When Tg was compared with UIE, median Tg of 12.4 µg/L (0.32–108.3 µg/L) and 22.5 µg/L (5.9–205.4 µg/L) were observed in children whose UIE >100 µg/L (n = 119) and children with UIE <100 µg/L (n = 36) respectively. The data were normally distributed for fT_3_ and fT_4_ but positively skewed for Tg and TSH, so natural log transformation was undertaken prior to analysis by one-way ANOVA. No significant association was found between fT_3_, fT_4_ and TSH. Significant association existed between Tg and UIE (p = 0.016), but post hoc analysis yielded no significant results.

### Thyroid function status

**Fig. 3:**
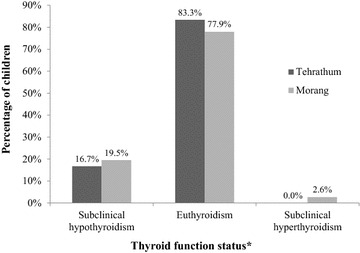
Percent distribution of Thyroid function status in primary school children of the Tehrathum and Morang districts. *Not statistically significant association between thyroid function categories and school children of Tehrathum and Morang districts [p = 0.311 (χ^2^ test)]

District wise distribution of sub-clinical hypothyroidism was found to be 16.7 % (n = 13) and 19.5 % (n = 15) in Tehrathum and Morang respectively (see Fig. 3). Subclinical hyperthyroidism was found in only two cases in Morang district. A total of 18.1 % of 155 cases, including 13.9 % (n = 11) boys and 22.4 % (n = 17) girls had subclinical hypothyroidism. Only two boys had subclinical hyperthyroidism. Fisher’s exact test shows that there was no significant association of thyroid dysfunction with sex (p = 0.153).

### Correlation of UIE, salt iodine content (SIC) and thyroid parameters

We applied Spearman’s rho correlation test for UIE, SIC, Tg and TSH whereas Pearson’s correlation test was used for fT_3_ and fT_4_ only. A significant positive correlation was found between UIE and SIC (r = 0.349, p < 0.0001), a negative correlation between UIE and Tg (r = −0.236, p = 0.003), and a negative correlation between UIE and TSH (r = −0.132, p = 0.102). A significant positive correlation was found between serum fT_3_ and fT_4_ (r = 0.284, p < 0.0001). No statistically significant correlations were found among fT_3_, fT_4_ and Tg. Serum Tg correlated positively with TSH (r = 0.285, p < 0.0001) and negatively with SIC (r = −0.222, p = 0.005). It is recognized that in spite of the highly significant correlations described above, the contribution to the total variance is small, leaving a high proportion of the variance unaccountable.

### Multiple linear regression analysis

We applied multiple linear regression analysis taking UIE as the dependent variable and age, gender, location and salt type as independent variables. Model fit was found statistically significant (F = 11.37, p < 0.001) explaining 6.7 % of the variation. The residual analysis showed error following normal distribution and having homogenous variance. This multivariate analysis showed that SAC of Morang district and using unpackaged salt having lower level of UIE (75.9 and 51.7 µg/L).

## Discussion

The WHO has estimated the worldwide prevalence of ID is 36.4 % in SAC leading to debilitating iodine deficiency disorders (IDDs). Improvements in ID have mainly been achieved through universal salt iodization (USI) and after implementation, careful monitoring of progress towards sustainable elimination of IDD is essential [15, 17, 29]. The main advantage of supplementation with iodized salt is that it is used by all sections of a community irrespective of social and economic status. Iodized salt is consumed in the same level throughout the year and the production is often confined to a few centers which means that processing can occur on a larger scale and with better controlled conditions [30]. Globally, iodine deficiency control programs using iodized salt have significantly reduced IDDs, but many countries still have weak or nonexistent national programs.

In Nepal, successive government administrations have shown concern about ID but have not been capable of implementing a successful USI program throughout Nepal. This has in part been due to major changes in political control and financial instability. Also, Nepal is made up of particularly diverse and challenging ecological regions (mountain, hill, and terai), comprised three administrative zones and 16 districts. Salt Trading Corporation (STC) Limited is the only authorized organization for the salt iodization and distribution of iodized salt in Nepal. The Government of India is aiding the iodization process. In Nepal, the recommended level of iodine is 50 mg iodine/kg of salt at the production level, 30 ppm at retail shop level and 15 ppm at household level. IDD status of Nepal can be improved by increasing the accessibility of the adequately iodized salt in all the areas including the remote places of hills and mountains [1]. In spite of this the government of Nepal has been engaged in surveys to study the incidence of ID since 1998. There are several indicators that may be used to determine the iodine status of individuals and a population and will be discussed in more detail below [16]. The WHO/UNICEF/ICCIDD [12] suggested that to be considered an iodine sufficient region, more than 50 % of the school children should have UIE level of (100 µg/L) or more, and not more than 20 % of samples should have UIE level of less than (50 µg/L). According to the Nepal National Survey and Impact study for IDDs in 2007, children having UIE <100 µg/L were 26.1 % in mountain, 18.9 % in hill and 9.1 % in terai in eastern Nepal [1]. In our focused study, children with UIE <100 µg/L were 10.5 % in Tehrathum (hill) and 9.1 % in Morang (terai). Comparison of our studies to the previous Nepal National Survey data showed lower values of ID in the Tehrathum hill region we studied, but showed agreement with the data from the Morang terai region.

The reason for the discrepancy is not known but it could be due to the focused coverage (only 7 clusters) of the Tehrathum region as we were unable to reach the most remote parts of the districts due to security and logistics problem, a recognized limitation of the study. This discrepancy could also be explained by the timing of the nationwide surveys. These were completed with school children in 1995, 2005 and 2007, with median UIE values of 143.8, 188.0 and 202.88 µg/L respectively [1, 9]. Our study could simply be showing the continued trend of sustainable improvement of UIE status of school children, which is supported by the previous studies [31–33]. These results provide an initial ‘snapshot’ of an iodine status in these populations.

**Fig. 4:**
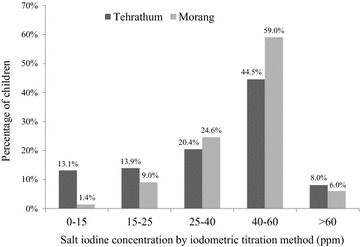
Percent distribution of salt iodine concentrations in primary school children in the Tehrathum and Morang districts

Interestingly, the UIE in 90.3 % of our population was higher than the WHO cut off point (100 µg/L) and is cause for concern because excessive iodine intake can be detrimental to health. Our study also showed interesting findings with almost 50 % having excessive UIE, though salt sample from their respective homes showed iodine concentrations within desired limits, with only approximately 7 % showing more than 60 ppm (See Fig. 4). This is of concern because elevated iodine can lead to induced hyperthyroidism and autoimmune thyroid disease. It is a challenging scenario to consider that the serious consequences of ID can coexist with those of excessive iodine. One potential source of high iodine values may be due to the eating habits of this particular population with frequent consumption of high-iodine uncooked instant noodles and flavor sachets in their lunch break. The high values for those using packaged salt are surprising. They exceed the levels of 15–40 ppm recommended by the WHO. One explanation is that when packaged salt, which is produced at a level of 50 ppm, is transported and stored, it then retains a much higher iodine level than originally anticipated. We observed this in our study, something that was similarly noted in a study undertaken in the Khumbu region of Nepal by Heydon et al. [34]. This illustrates further how regular sampling of iodine status and basic education about nutritional supplements are necessary so that the appropriate action taken to increase or decrease iodine intake.

This leads directly into the consideration of the type of testing that should be conducted to assess the iodine status of individuals and populations accurately. Measurement of serum Tg was recommended by the WHO in 1994 as a more sensitive indicator for thyroid cell mass and as a better monitor for iodine status than the other three indicators, UIE, Total goiter rate (TGR), and serum TSH [35]. The WHO also suggested that a median Tg concentration of <10 µg/L in a population indicated iodine sufficiency [35]. However, data confirming Tg cutoff value were limited.

In a recent study by Zimmerman et al., the cut off point for median Tg of 13 µg/L was proposed using clusters with median UIE in the range of 100–299 µg/L, after CRM-457 standardization, which indicates iodine sufficiency in a population of school-aged children [28]. There were technical problems in measurements of Tg including large interassay variability and lack of reference data for Tg in healthy, iodine-sufficient school age children [28, 36].

The median Tg in this study was lower than the cut-off point estimated by the manufacturer (50 µg/L) and Zimmermann et al. [27] as reported in the results section. We propose that the Tg values obtained in this study could be ranked as iodine sufficiency regions if the cut-off of 50 µg/L is applied. The UIE and SIC estimation in this study suggests, with the limitation of single spot sample, an iodine sufficiency region comparable with the significant decrease of Tg level. Thus, the median Tg concentration could be used as a suitable indicator for monitoring iodine status.

Our focused study of UIE levels in two districts in Nepal gave a reasonable representation of the region and it is unlikely that there are large groups of the population where iodine deficiency has not been assessed. Our results agree with previous trends seen in national surveys showing that iodized salt programs are steadily improving the iodine deficiency problems that plagued Nepal and other developing countries in recent times.

### Limitations

The present study has some limitations. Firstly to generalize the result to whole populations, a larger sample size of school children is required that would need considerably more time and resource. Secondly, we have not assessed Anti Tg Antibody, which may interfere with the result of serum Tg and we have also not measured Anti thyroid Antibody, which can help to differentiate the etiology of subclinical hypothyroidism. In both these cases, it was due to limited resources.

## Conclusion

There has been considerable progress in the last decade towards the elimination of iodine deficiency in Nepal reflecting the validity of the WHO strategy of salt iodization programs. Our focused study on two districts of Nepal, one hill district (Tehrathum) and one terai district (Morang), showed continued improvement in the iodine status of the population based on UIE levels suggesting that there is increasing lower ID in these regions. Also, promising results from our study found that the proportion of households consuming adequately iodized salt have reached target levels of >90 % in the Morang district and are steadily approaching those levels in the Tehrathum district. These results reflects the efforts made by countries to implement effective IDD control programs and is evidence of the successful collaboration and partnership between IDD control agencies, mostly the health authorities and the salt industries.

The results of our study also uncovered an unexpected and complicating issue of surprisingly high levels of iodine excess in the same population with a proportion of people showing low iodine status. This complicates a standardized intervention strategy that only focuses on salt iodization supplementation and suggests that an intervention approach needs to be a more dynamic tailored strategy. This new strategy would have to include rapid testing, timely intervention, and basic nutritional education specific to the population. This will require coordination between non-government and government institutions and every effort needs to be made to ensure that those involved continue to cover at-risk populations. Even with the substantial progress that has been made, the challenge now is to improve the quality of the data in order to generate appropriate and timely interventions and to track progress more accurately and rapidly.

## Abbreviations

UIE: urinary iodine excretion
SIC: salt iodine content
SAC: school-aged children
ID: iodine deficiency
IDD: iodine deficiency disorder
TSH: thyroid stimulating hormone
fT3: free triiodothyronine
fT4: free thyroxine
Tg: serum thyroglobulin

## References

[1] Ministry of Health and Population, Department of Health Services, Government of India and Alliance Nepal. National Survey and Impact Study for Iodine Deficiency Disorders (IDD) and availability of iodized salt in Nepal; 2007.

[2] Diaza J, Cagigas A, Rodrguez R (2003). Micronutrient deficiencies in developing and affluent countries Africa.

[3] Zimmermann MB, Jooste PL, Pandav CS (2008). Iodine-deficiency disorders. Lancet.

[4] De Benoist B, Andersson M, Egli IM, El Bahi T, Allen H, World Health Organization. Dept. of Nutrition for Health and Development (2004). Iodine status worldwide: WHO Global Database on Iodine Deficiency.

[5] Zimmermann M. Technical brief: key barriers to global iodine deficiency disorder control: a summary. 2007. p. 1–10.

[6] Anderson GW, Schoonover C, Jones S (2003). Control of thyroid hormone action in the developing rat brain. Thyroid..

[7] Arthur JR, Beckett G. Thyroid function. 1999;55:658–68 10.1258/0007142991902538 10746354

[8] Stephenson T, Arora A, Tolley N, Tuttle R (2010). Pathological spectrum of thyroid disease. A practical manual of thyroid and parathyroid disease.

[9] Ministry of Health and Population, Child Health Division, Micronutrient Initiative, New ERA. Nepal iodine deficiency disorders status survey; 2005.

[10] Ristic-Medic D, Piskackova Z, Hooper L, Ruprich J, Casgrain A, Ashton K (2009). Methods of assessment of iodine status in humans: a systematic review. Am J Clin Nutr.

[11] Delange F, Burgi H, Chen ZP, Dunn JT (2002). World status of monitoring iodine deficiency disorders control programs. Thyroid..

[12] World Health Organization, International Council for Control of Iodine Deficiency Disorders, UNICEF (2001). Assessment of iodine deficiency disorders and monitoring their elimination: a guide for programme managers.

[13] Vejbjerg P, Knudsen N, Perrild H, Laurberg P, Andersen S, Rasmussen LB (2009). Estimation of iodine intake from various urinary iodine measurements in population studies. Thyroid..

[14] Zimmermann MB, Andersson M (2012). Assessment of iodine nutrition in populations: past, present, and future. Nutr Rev.

[15] Bonofiglio D, Catalano S, Perri A, Baldini MP, Marsico S, Tagarelli A (2009). Beneficial effects of iodized salt prophylaxis on thyroid volume in an iodine deficient area of southern Italy. Clin Endocrinol.

[16] World Health Organization (2007). Assessment of iodine deficiency disorders and monitoring their elimination: a guide for programme managers.

[17] Zimmermann MB, Moretti D, Chaouki N, Torresani T (2003). Development of a dried whole-blood spot thyroglobulin assay and its evaluation as an indicator of thyroid status in goitrous children receiving iodized salt. Am J Clin Nutr.

[18] Buchinger W, Lorenz-Wawschinek O, Semlitsch G, Langsteger W, Binter G, Bonelli RM (1997). Thyrotropin and thyroglobulin as an index of optimal iodine intake: correlation with iodine excretion of 39,913 euthyroid patients. Thyroid..

[19] Delange F, Braverman LE, Utiger RD, Werner SC, Ingbar SH (2000). Werner & Ingbar’s the thyroid: a fundamental and clinical text. Iodine deficiency.

[20] Delange F, Camus M, Ermans AM (1972). Circulating thyroid hormones in endemic goiter. J Clin Endocrinol Metab.

[21] Joshi AB, Banjara MR, Bhatta LR, Rikimaru T, Jimba M (2007). Insufficient level of iodine content in household powder salt in Nepal. Nepal Med Coll J..

[22] Gelal B, Baral N (2010). Moving towards the sustainable elimination of IDD in Nepal. IDD Newsl..

[23] Ohashi T, Yamaki M, Pandav CS, Karmarkar MG, Irie M (2000). Simple microplate method for determination of urinary iodine. Clin Chem.

[24] Genesis Diagnostics Ltd. Thyroglobulin antigen ELISA Kit: quantitative assay for the detection of human thyroglobulin antigen.

[25] Biswas AB, Das DK, Chakraborty I, Biswas AK, Sharma PK, Biswas R (2014). Goiter prevalence, urinary iodine, and salt iodization level in sub-Himalayan Darjeeling district of West Bengal, India. Indian J Public Health.

[26] Sridhar P, Kamala C (2014). Iodine status and prevalence of Goitre in school going children in rural area. J Clin Diagn Res.

[27] Zimmermann MB, de Benoist B, Corigliano S, Jooste PL, Molinari L, Moosa K (2006). Assessment of iodine status using dried blood spot thyroglobulin: development of reference material and establishment of an international reference range in iodine-sufficient children. J Clin Endocrinol Metab.

[28] Zimmermann MB, Aeberli I, Andersson M, Assey V, Yorg JAJ, Jooste P (2013). Thyroglobulin is a sensitive measure of both deficient and excess iodine intakes in children and indicates no adverse effects on thyroid function in the UIC range of 100–299 μg/l: a UNICEF/ICCIDD study group report. J Clin Endocrinol Metab.

[29] de Benoist B, McLean E, Andersson M, Rogers L (2008). Iodine deficiency in 2007: global progress since 2003. Food Nutr Bull.

[30] Baral N, Lamsal M, Koner BC, Koirala S (2002). Thyroid dysfunction in eastern Nepal. Southeast Asian J Trop Med Public Health.

[31] Baral N, Ramaprasad C, Lamsal M, Koner BC, Koirala S (1999). Assay of iodine deficiency status in three ecological regions of Nepal by a microdigestion method. Southeast Asian J Trop Med Public Health.

[32] Gelal B, Chaudhari RK, Nepal AK, Sah GS, Lamsal M, Brodie DA (2011). Iodine deficiency disorders among primary school children in eastern Nepal. Indian J Pediatrics..

[33] Gelal B, Aryal M, Lal Das B, Bhatta B, Lamsal M, Baral N (2009). Assessment of iodine nutrition status among school age children of Nepal by urinary iodine assay. Southeast Asian J Trop Med Public Health.

[34] Heydon EE, Thomson CD, Mann J, Williams SM, Skeaff SA, Sherpa KT (2009). Iodine status in a Sherpa community in a village of the Khumbu region of Nepal. Public Health Nutr.

[35] World Health Organization, International Council for Control of Iodine Deficiency Disorders, UNICEF (1994). Indicators for assessing iodine deficiency disorders and their control through salt iodization. Micronutrient series.

[36] Spencer CA, Wang CC (1995). Thyroglobulin measurement. Techniques, clinical benefits, and pitfalls. Endocrinol Metab Clin North Am.

